# COMMUNITY ADVISORY COUNCILS: AN ENGAGEMENT STRATEGY FOR PATIENT-CENTERED LEARNING

**DOI:** 10.1093/geroni/igad104.1575

**Published:** 2023-12-21

**Authors:** Jennifer Severance

**Affiliations:** University of North Texas Health Science Center, Fort Worth, Texas, United States

## Abstract

Advisory councils provide a forum for patients and families to partner with health care systems and share experiences, perspectives, ideas, feedback, and recommendations that can improve both the patient experience and health care outcomes. Research shows that patient partnerships fostered through advisory councils can also positively impact health professions education and training by supporting patient-centered learning. With a goal to promote collaboration among community stakeholders in the delivery of geriatrics education and training, a Geriatric Workforce Enhancement Program formed a Community Advisory Council. A project team developed the council goals, structure, application and recruitment processes, and facilitation methods. Over two years, a total of 27 older adults, caregivers and community advocates participated in the advisory council both online and in-person. Council members completed an orientation session and met four times over a twelve-month period. At each meeting, faculty prepared two to four open-ended discussion questions regarding knowledge, skills, and attitudes relevant to the nursing home workforce, student education, continuing education, and community outreach. Responses were collected through paper surveys and online forms. Thematic analysis of qualitative responses identified common themes that were shared with education and clinical leadership to inform and improve learning experiences, health care processes, and council engagement. Presenters will describe the council’s structure and composition as well as recruitment, facilitation, and evaluation methods, and lessons learned. This project demonstrates a replicable model of evaluating education that involves older adults, families and community in co-creating quality learning and patient experiences.

